# Cost-Effectiveness of Adolescent Pertussis Vaccination for The Netherlands: Using an Individual-Based Dynamic Model

**DOI:** 10.1371/journal.pone.0013392

**Published:** 2010-10-15

**Authors:** Robin de Vries, Mirjam Kretzschmar, Joop F. P. Schellekens, Florens G. A. Versteegh, Tjalke A. Westra, John J. Roord, Maarten J. Postma

**Affiliations:** 1 Unit of Pharmacoepidemiology and Pharmacoeconomics, Department of Pharmacy, University of Groningen, Groningen, The Netherlands; 2 Centre for Infectious Disease Control, National Institute for Public Health and the Environment (RIVM), Bilthoven, The Netherlands; 3 Julius Centre for Health Sciences and Primary Care, University Medical Centre Utrecht, Utrecht, The Netherlands; 4 Laboratory for Infectious Diseases, Groningen, The Netherlands; 5 Department of Pediatrics, Groene Hart Ziekenhuis, Gouda, The Netherlands; 6 Department of Pediatrics, VU University Medical Center, Amsterdam, The Netherlands; Cochrane Acute Respiratory Infections Group, Italy

## Abstract

**Background:**

Despite widespread immunization programs, a clear increase in pertussis incidence is apparent in many developed countries during the last decades. Consequently, additional immunization strategies are considered to reduce the burden of disease. The aim of this study is to design an individual-based stochastic dynamic framework to model pertussis transmission in the population in order to predict the epidemiologic and economic consequences of the implementation of universal booster vaccination programs. Using this framework, we estimate the cost-effectiveness of universal adolescent pertussis booster vaccination at the age of 12 years in the Netherlands.

**Methods/Principal Findings:**

We designed a discrete event simulation (DES) model to predict the epidemiological and economic consequences of implementing universal adolescent booster vaccination. We used national age-specific notification data over the period 1996–2000—corrected for underreporting—to calibrate the model assuming a steady state situation. Subsequently, booster vaccination was introduced. Input parameters of the model were derived from literature, national data sources (e.g. costing data, incidence and hospitalization data) and expert opinions. As there is no consensus on the duration of immunity acquired by natural infection, we considered two scenarios for this duration of protection (i.e. 8 and 15 years). In both scenarios, total pertussis incidence decreased as a result of adolescent vaccination. From a societal perspective, the cost-effectiveness was estimated at €4418/QALY (range: 3205–6364 € per QALY) and €6371/QALY (range: 4139–9549 € per QALY) for the 8- and 15-year protection scenarios, respectively. Sensitivity analyses revealed that the outcomes are most sensitive to the quality of life weights used for pertussis disease.

**Conclusions/Significance:**

To our knowledge we designed the first individual-based dynamic framework to model pertussis transmission in the population. This study indicates that adolescent pertussis vaccination is likely to be a cost-effective intervention for The Netherlands. The model is suited to investigate further pertussis booster vaccination strategies.

## Introduction

Pertussis (whooping cough) is a highly contagious infection of the respiratory tract. It is caused by the bacteria *Bordetella pertussis*, and occasionally, *Bordetella parapertussis*. Before the introduction of vaccination programs, pertussis was a main cause of child morbidity and mortality in developed countries. After the introduction of routine vaccination programs the incidence of pertussis decreased to very low levels. However, during the past decade the incidence has again shown an increasing trend in many developed countries with a shift in the incidence towards older age groups [1].

Despite a high vaccination coverage (≈96%) a clear increase in the incidence of pertussis was also apparent in the Netherlands from 1996 onwards [2]–[4]. This increase is most marked in adolescents and adults. This is of concern as adolescents and adults have been identified as a major source of transmission of pertussis to very young yet unimmunized and only partly immunized infants, in whom pertussis causes serious morbidity and mortality [5], [6]. As pertussis infections often go by unnoticed in adolescents and adults, partly because the disease is often misdiagnosed in these age groups by clinicians and partly as many infections remain asymptomatic, the actual incidence in this age group probably is much larger due to underreporting [7], [8]. Although pertussis is not generally recognized as a serious problem beyond childhood, it can cause significant morbidity and costs in adolescents and adults with productivity losses comprising the largest costs [9], [10].

In recent years, acellular vaccines have been replacing whole cell vaccines in many developed countries [1], [11]. These acellular vaccines are less reactogenic, improving their safety profile. In the Netherlands, until 2001, the vaccination schedule consisted of four doses of the whole cell pertussis vaccine (currently at 2, 3, 4 months of age and a booster vaccination at 11 months of age). From 2001 onwards, an additional booster vaccination (acellular vaccine) is given to four year old children to reduce the incidence. Moreover, from 2005, the four whole cell vaccinations given within the first year of life have been replaced by the acellular vaccine [12].

Vaccine-induced immunity is known to be of relatively short duration (∼8 years), after either immunization with whole cell or acellular vaccines [13], [14]. Considering the fact that thus adolescents and adults are a major reservoir for pertussis transmission to very young infants, addition of immunization strategies to the current childhood program to decrease the incidence in adolescents or adults may be considered. A decrease in the pertussis incidence in adolescents or adults may be expected to lead to an increase in herd immunity that in turn indirectly results in an increased protection of young vulnerable infants [5], [15]. Currently, several countries (e.g. Germany, France, Canada, Australia) have already incorporated an adolescent booster dose (acellular vaccine) into their immunization program [1], [16].

The main goal of this paper is to design an individual-based stochastic dynamic framework to model the pertussis transmission in the population in order to predict the epidemiologic and economic consequences of the implementation of universal booster vaccination programs. Using this framework, we currently estimate the cost-effectiveness of universal adolescent pertussis booster vaccination at the age of 12 years in the Netherlands.

## Methods

In this section we elaborate on the individual-based dynamic model and the data used for the cost-effectiveness analysis. In general, the model consists of a dynamic epidemiologic part and an economic part. In particular, the epidemiologic part can be used for estimating the impact of an universal booster vaccination strategy on the incidence and prevalence of pertussis in the population. Specifically, here, it was used for estimating the epidemiologic consequences of adolescent booster vaccination in the Netherlands. The economic part addresses the averted complications, associated costs and the cost analysis of the adolescent booster vaccination. Both models were linked by using the output of the epidemiologic part as input for the economic analysis to estimate cost-effectiveness.

### Dynamic Epidemiologic Model

**Figure 1:**
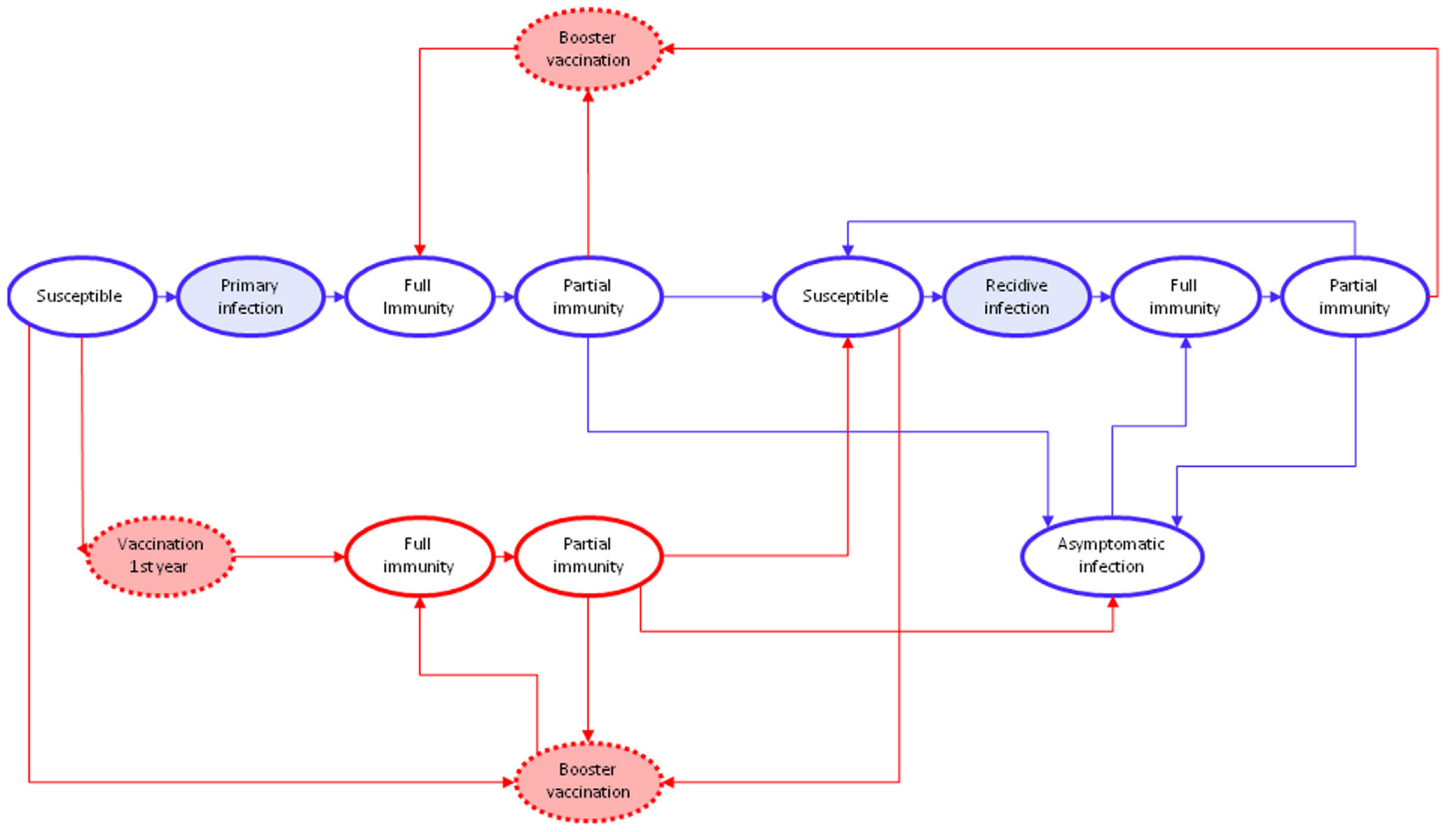
Schematic representation of the possible pathways within the pertussis DES (Discrete Event Simulation) model. Red circles and red lines indicate events and pathways associated with vaccination, respectively; dashed circles indicate events where no time is actually involved; and shaded circles indicate events where resources are consumed and therefore costs are included.

We designed a discrete event simulation (DES) model to model pertussis transmission in the population [17]–[19]. Contrary to deterministic SIR (Susceptible-Infected-Recovered) models, which are often used to model infectious diseases, within a DES-model individual persons are modeled rather than cohorts [17]–[19], [20]. Furthermore, a fundamental aspect of DES is time which advances in ‘discrete’ jumps; for every individual the time until the next event happens is determined and the simulation proceeds along a chain of events [17]–[19]. A schematic representation of the possible individual pathways within the designed pertussis DES-model is shown in Figure 1.

We developed a model that distinguishes between three types of infections: infections in immunologically naive individuals (i.e. primary infections), infections in individuals whose immune system has been primed before by vaccination or infection (i.e. recidive infections), and asymptomatic infections (note that all primary and recidive infections were assumed to be symptomatic). All individuals are born susceptible after which they run the risk of acquiring a pertussis infection after contact with an infectious person. After infection, individuals recover and become immune. Immunity wanes over time. We divided immunity into two types: full immunity (i.e. immunity against infection and disease), and partial immunity (i.e. immunity against disease only). In particular, although a partial immune individual is able to acquire an infection and subsequently transmit the pathogen to other individuals in the population, the partial immune individual will not become ill (i.e. only experiences asymptomatic infection). Furthermore, we assumed that if a partial immune, or susceptible, individual comes into contact with the pathogen by either vaccination or infection, the person will become full immune again through this next priming of the immune system. The DES-model was built in Arena® [21].

### Model Parameters

Although the duration of the infectious period is not precisely known and probably varies widely, it likely depends on the severity of the disease. Based on expert opinions, we assumed that the mean period which an individual with pertussis is infectious lasts 4 weeks for primary infected cases, 3 weeks for recidive infected cases and 1 week for asymptomatic cases.

We assumed that childhood vaccination as well as adolescent vaccination has influence on the level of immunity of both partial immune and susceptible individuals. As a result of vaccination, both groups were assumed to become immune against both disease and infection (Figure 1). The vaccination coverage in the Netherlands is about 96% for infants and the same was assumed for adolescents. In the model we divided the vaccination scheme into three parts: (i) the vaccinations at 2, 3, 4 and 11 months of age; (ii) the booster dose at 4 years of age; and (iii) a booster in adolescents at 12 years of age. In reality immunity after vaccination is gradually built up after each vaccination dose within the first year of life. However, for simplicity, with regard to the immunization within the first year we assumed that a certain fraction of the population (i.e. coverage×efficacy) is effectively protected precisely after 4 months (i.e. 1 month after the second vaccination at 3 months of age).

Before the implementation of the acellular booster vaccination at the age of 4 years, the efficacy of the whole cell pertussis vaccine used in the Netherlands was estimated at 89% [2], [22]–[25]. This estimate was based on observational data from the Netherlands, in particular national notification reports, during the period when in the Netherlands a locally-produced whole-cell vaccine was used (manufacturer: National Institute of Public Health/Netherlands Vaccine Institute). Since 2001, acellular vaccines have been introduced in the Dutch Immunization Program, based on an advice by the Dutch Health Council that efficacy of these vaccines would not be inferior to those of the whole-cell vaccine and a superior safety profile of the acellular products [26]. In particular, 5-component acellular pertussis vaccines are currently used in the Netherlands for infants (Pediacel^(R)^ and Infanrix-hexa^(R)^) and for boostering at the age of 4 (Infanrix-IPV^(R)^). These multi-component (≥3 components) vaccines have a higher efficacy than one- and two-component acellular vaccines and have not been assessed inferior to the best whole cell vaccines on a statistically significant level [27], [28]. Based on clinical trial data the efficacy of an acellular adolescent booster vaccine for adolescents was also estimated at a quite similar level [29]–[31]. Therefore, in the model we assumed the same efficacy at 89% for vaccination within the first year, booster vaccination at 4 years of age and booster vaccination at 12 years of age. The duration of protection after vaccination is discussed below (*see* Scenarios section).

In general, the most important term in a dynamic model would be the so-called force of infection (FOI). The FOI denotes the rate at which susceptible individuals acquire an infection [20]. Here we used the method developed by van Boven et al [23] to estimate the age-dependent FOI from age-specific incidence data. The advantage of that method is that the estimation procedure is based on the underlying model structure. Furthermore, it is possible to take the waning immunity and different types of infection consistently into account. For the estimation of the age-specific FOI we had to assume a stable age distribution and an endemic equilibrium (i.e. steady state). We divided the population in 86 age classes; 74 yearly classes (1–74 years) and the first year separated into monthly classes (0–11 months). We assumed an endemic equilibrium from 1996 to 2000. Therefore, we averaged the case notification data from the Netherlands over those years to obtain the age-specific steady state incidences and prevalences [32].

Subsequently, these numbers were corrected for underreporting. Previously, de Melker et al estimated that, in the Netherlands, the true incidence was approximately 660 times higher than the reported incidence for children and adults [33]. We used the age specific ratios of estimated numbers to notified numbers for correcting the age-specific incidences [33]. We note that in reality there probably was no endemic equilibrium from 1996 to 2000 [32], but for correctly analyzing the effects of different immunization strategies a steady state situation has to be assumed in order to discard the time dependence in the incidence and prevalence. The set of differential equations used for estimating the age-specific FOI is given in Appendix S1. For a full description of the methodology we refer to van Boven et al [23].

The FOI is a function of the number of infectious individuals at a given point in time, the contact function and the transmission coefficients. Here, the age specific FOI is given by:
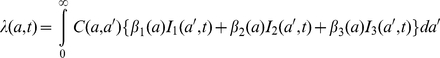
Where I_1_, I_2_ and I_3_ are the prevalences of primary infected, recidive infected and asymptomatically infected individuals, respectively. The contact function (C(a,a′)) is a 86×86 matrix representing the number of contacts between an individual in age-group *a* with an individual in age-group *a′* per unit of time. We used the contact matrix for respiratory diseases for the Dutch population estimated by Wallinga et al [34], [35]. The transmission coefficient (β) represents the probability that a contact between a susceptible individual of age *a* with an infectious individual of age *a′* leads to transmission. We assumed different transmission probabilities concerning the different types of infection, with β_1_(a)∶β_2_(a)∶β_3_(a) = 1∶0.7∶0.05. I.e. the more severe the disease the higher the probability of transmission. Furthermore, we assumed that the transmission probability depends on the age of the susceptible individual. The age-specific transmission parameters were estimated from the estimated age specific FOI, the contact matrix and the age specific steady state prevalence. Ergo, the transmission coefficients were used to calibrate the model on the assumed age-specific steady state prevalences.

### Scenarios & Calculations

The exact duration of immunity acquired by natural infection is not known. A recent review on the duration of immunity after natural infection revealed estimates of protection varying from 4 to 20 years [13]. Accordingly, we considered two scenarios that differ in the duration of immunity after a natural pertussis infection. In scenario 1 we assumed that immunity on average wanes after 15 years (R_n_ = 15), where individuals were fully and partially protected for 2 and 13 years, respectively. In scenario 2, we assumed that immunity on average wanes after 8 years (R_n_ = 8), where individuals were fully and partially protected for 2 and 6 years, respectively. The duration of immunity acquired by vaccination did not differ between both scenarios at 8 years.

For both scenarios the situation in which a pertussis adolescent booster vaccination would be implemented was compared to the current situation without adolescent booster vaccination. Simulations were performed for a population of 150,000 individuals with a uniform age distribution (i.e. the age classes were equally sized). The population size was stabilized with newborns who enter the model exactly balancing the people who leave the model due to death. Type 1 mortality was assumed as this is generally a reasonable approximation for developed countries [20]. In particular, we assumed that everybody lives to 75 years of age and dies thereafter.

To mimic reality, at the start of the simulation (t = 0) a booster vaccination at the age of 4 years is implemented in the model. Subsequently, for assessing the impact of an adolescent booster dose, after 10 years (t = 10) an extra vaccination at the age of 12 years is implemented in the model. Hereafter, a period of another 15 year was chosen for analyzing the impact of the adolescent booster vaccination on the pertussis incidence and prevalence in the population. This means that a total time horizon of 25 years was used. Obviously, when assessing the outcomes over 25 years of the current situation, no booster vaccination at t = 10 was implemented in the model. The parameter values used in the stochastic model for both scenarios are shown in Table 1. Because the model is stochastic, multiple simulation runs were performed for both scenarios in order to capture the uncertainty in the results.

**Table 1:** Variables used for both scenarios in the epidemiologic discrete event simulation model.

Variable	Value	Probability* (daily)	References
*Infectious period*			
I_1_	4 weeks	0.035	Expert panel
I_2_	3 weeks	0.047	Expert panel
I_3_	1 week	0.133	Expert panel
*Loss of immunity*			
Scenario 1			
Loss of full immunity rate after infection	2 years	0.00137	Expert panel, [13], [14]
Loss of partial immunity rate after infection	13 years	0.00021	Expert panel, [13], [14]
Loss of full immunity rate after vaccination	2 years	0.00137	Expert panel, [13], [14]
Loss of partial immunity rate after vaccination	6 years	0.00046	Expert panel, [13], [14]
Scenario 2			
Loss of full immunity rate after infection	2 years	0.00137	Expert panel, [13], [14]
Loss of partial immunity rate after infection	6 years	0.00046	Expert panel, [13], [14]
Loss of full immunity rate after vaccination	2 years	0.00137	Expert panel, [13], [14]
Loss of partial immunity rate after vaccination	6 years	0.00046	Expert panel, [13], [14]

*The daily probability for an individual to lose infectiousness or immunity.

The expert panel consisted of: M. Kretzschmar, J.F.P. Schellekens, F.G.A. Versteegh, J.J. Roord and J.T. Poolman.

### Economic Model

The output of the dynamic model, in terms of annual primary, recidive and asymptomatic infections, was used to estimate the economic consequences of the implementation of the adolescent booster vaccination program. In particular, the different types of infections were linked to associated resource use and subsequent costs. We considered the following costs: (i) vaccination costs (i.e. vaccine price and administration costs); (ii) direct medical costs associated with diagnosis, therapy and possible length of hospital stay; and (iii) indirect costs due to productivity losses. All costs were reported in €'s at 2008 price levels. If there were no cost estimates available for 2008 we converted them to 2008 prices using gross domestic product deflators [36].

The vaccination costs included the vaccine price and the administration costs. One dose of the acellular pertussis vaccine registered for the use in infants costs €18.30 [37]. As the price for the accellular adolescent booster vaccine was not available at that time we assumed a price of €18.30 for the adolescent booster vaccine as well. Furthermore, the vaccine administration costs were assumed at €6. Summarizing, the total vaccination costs aggregated at €24.30 per individual.

The direct medical costs included costs associated with diagnostic procedures, antibiotic treatment and hospital stay. We assumed that these direct medical costs differ between notified and unnotified cases. For estimating the age specific proportion of notified cases per type of infection (i.e. I_1_, I_2_ and I_3_) the age specific steady state ratios I_1_∶I_2_∶I_3_ (where underreporting was taken into account) together with the age specific notification data (from 1996 to 2000) were used. First, we assumed that notified persons suffered from a primary pertussis infection. Subsequently, if the incidence of notified cases exceeded the incidence of primary infections, the remainder was assumed to suffer from a recidive infection. Obviously, all asymptomatic infections were assumed to be unnotified. The age- and infection-type specific reporting rates are shown in Appendix S1 (Table B1). Furthermore, the age- and infection-type specific hospitalization rates were estimated using data on pertussis related hospital admissions obtained from the Dutch Prismant-database [38]. The estimation procedure of the hospitalization rates was similar to that of the reporting rates; we assumed that hospitalized persons suffered from a primary pertussis infection and, if the incidence of hospitalizations exceeded the incidence of primary infections, the remainder was assumed to suffer from a recidive infection.

The exact hospitalization rates are shown in Appendix S1 (Table C1). Furthermore, national data on pediatric intensive care stays revealed that 5.93% and 1.82% of the pertussis related hospital admissions lead to intensive care stays for infants at the age of 0 and 1 years respectively (data obtained from the national PICU registry). The age-specific mean lengths of stay of the pertussis-related hospital admissions are shown in Appendix S1 (Table C2). The average costs of a pediatric intensive care stay were assumed at €5000 per admission plus an extra cost of €1500 per day (data obtained from the VU University Medical Centre in Amsterdam, the Netherlands). The average daily costs for a regular (i.e. standard care) stay were estimated at €367 [39].

Of the notified pertussis cases who were not hospitalized a certain fraction was assumed to be treated ambulatory by a medical specialist (e.g. pediatrician or lung specialist). As young infants and elderly are most likely to be treated ambulatory we assumed that these fractions were age dependent (Appendix S1; Table C3). Resources used by all persons treated in or admitted to the hospital were assumed to include: (i) 2 diagnostic laboratory tests (which contained 1 polymerase chain reaction (PCR) test and 1 serology test); (ii) chest radiography; (iii) CRP (C-reactive protein); (iv) blood concentration determination (e.g. leukocyte count); and (v) antibiotic treatment with azithromycin. We assumed that those persons additionally had on average one general practitioner (GP) visit and three specialist visits. Furthermore, the remainder of the notified cases was assumed to incur 2 GP visits together with one course of azithromycin treatment. For those we assumed that 83%, 4% and 13% were diagnosed by means of serology, culture and PCR (data obtained from Infectious Diseases Laboratory Groningen, the Netherlands). All medical costs are shown in Table 2.

**Table 2:** Direct medical costs used as inputs for the economic model (2008 euros).

Variable	Costs	References
*Diagnostic procedures*		
Chest radiography	48.49	[39]
C-reactive protein	6.09	[38]
Blood concentration determination	9.74	[38]
PCR	99.13	[38]
Serology	43.38	[38]
Culture	15.00	[38]
*Treatment*		
Azithromycine for adolescents and adults	8.20	[37]
Azithromycine for infants <12 yrs	2.44/kg*	[37], [42]
Prescription fee (pharmacist)	6.10	[37]
Consult general practitioner	21.75	[39]
Consult medical specialist	52.89#	[39]
*Hospital stay*		
Standard care day	387.75	[39]
Pediatric intensive care day	1500	[hospital†]
Pediatric intensive care admission costs (one-off)	5000	[hospital†]

† VU University Medical Centre Amsterdam, Netherlands.

*We calculated age-specific costs by using national data on age-specific weights [39].

# A consult was assumed to last 30 minutes.

Our analysis was performed using the societal perspective; both the direct medical costs (irrespective of reimbursement issues) and the indirect costs of production losses were included in the analysis. Tormans et al reported, based on replies from pediatricians, that one of the parents would be absent of work for approximately 10 days while a child was suffering from pertussis [40]. We used this estimate for children below the age of one, irrespective of infection type and notification status. Conservatively, the mother was assumed to care for the child (or adolescent). Moreover, in general, if a person is hospitalized, the mean length of stay period was added to the estimate. However, we did not include productivity losses for children aged 0 to 3 months because of the maternity leave entitled to new mothers. For notified children aged 1 to 9 years we assumed a work absence of 6 days by one of the parents as reported by Lee et al [41]. For this group (and adolescents and adults) we assumed that unreported cases result in half of the productivity losses. The time missed from work due to a pertussis infection in adolescents and adults has been estimated by Lee et al [9]. For adolescents (10–19 years) they estimated a mean work loss of 1.03 days for one of the parents. However, for adolescents aged 15 to 17 years we assumed a work loss of 2.65 days for those adolescents that work themselves [9]. Finally, we assumed that adults miss a mean of 5.98 days of work [9]. Note that we did not distinguish productivity losses between primary and recidive infections as the estimates extracted from the literature apply to reported cases regardless of infection type [9], [40], [41]. Obviously, we did not include productivity losses for asymptomatic infected persons in the analysis.

Subsequently, the friction cost method was used to monetarily value the indirect costs [42], [43]. Here, we took both the national unemployment and the elasticity of absence from work related to productivity (i.e. 0.8 for the Netherlands) into account [39], [44]. All age-specific estimates of productivity losses and associated indirect costs are shown in Table 3.

**Table 3:** Productivity losses (days) and associated indirect costs (€2008) per pertussis case.

Age*	Hospitalized	Costs	Reported	Costs	Unreported	Costs
*0–3 m*	0	0	0	0	0	0
*3–11 m*	17.98	1095	10.00	609	5.00	304
*1 yr*	10.05	612	6.00	365	3.00	183
*2 yrs*	9.80	597	6.00	365	3.00	183
*3 yrs*	9.36	570	6.00	365	3.00	183
*4–9 yrs*	10.08	523	6.00	311	3.00	156
*10–14 yrs*	5.32	276	1.03	53	0.52	27
*15–17 yrs*	7.15	146	2.65	54	1.33	27
*18–19 yrs*	10.48	213	5.98	122	2.99	61
*20–24 yrs*	10.68	217	5.98	122	2.99	61
*25–34 yrs*	10.68	875	5.98	490	2.99	245
*35–44 yrs*	10.68	988	5.98	553	2.99	277
*45–54 yrs*	10.68	1004	5.98	562	2.99	281
*55–64 yrs*	10.68	713	5.98	399	2.99	200
*65+ yrs*	0	0	0	0	0	0

*For children aged 0 to 14 years the productivity losses refer to the mother.

### Cost-Effectiveness Analysis

The incremental cost-effectiveness was estimated for of a pertussis booster vaccination program aimed at adolescents compared to the current situation (i.e. no adolescent pertussis vaccination). Cost-effectiveness was expressed as net costs per quality adjusted life year (QALY). Hence, we linked quality weights to the complications related to pertussis infections. Most of the utilities used here were extracted from the only published study on preferences in adults or parents of children with pertussis illness [45]. If no utilities were available, reasonable figures were assumed (Table 4). In the baseline analysis, we assumed that hospitalized, reported and unreported cases suffer from severe cough, moderate cough and mild cough, respectively. Pertussis illness in infants aged <1 years of age was always assumed severe. Estimates for the mean durations of pertussis illness were obtained from available data [45], [46]: (i) 80 days for infants aged <1 year; (ii) 56 days for children aged 1–9 years; (iii) 74 days for adolescents; and (iv) 87 days for adults. As the severity of pertussis disease wanes over time we assumed that adults did not have be off work the full period, either to take care of their ill child or because of their own illness. Consequently, the full period over which quality of life losses occur does not reflect the period of productivity losses. According to the Dutch guidelines future health outcomes and future costs were discounted at 1.5% and 4%, respectively [47].

**Table 4:** Age-specific decrements in utility related to pertussis illness.

Age	Mild cough	Moderate cough	Sever cough
0 yr	-	-	0.42 [46]
1–3 yrs	0.20 [Assumed]	0.28 [Assumed]	0.39 [Assumed]
4–9 yrs	0.175 [Assumed]	0.25 [Assumed]	0.36 [Assumed]
10–17 yrs	0.15 [Assumed]	0.22 [45]	0.33 [46]
18–59 yrs	0.10 [46]	0.15 [45]	0.19 [46]
60+ yrs	0.10 [46]	0.15 [45]	0.19 [46]

References are given between brackets.

### Sensitivity analysis

To further test the robustness of the outcomes we performed a univariate sensitivity analysis on various parameters in the economic model. For example, the inclusion of universal pertussis vaccination in the Dutch national immunization program would probably result in a decline in vaccine price due to large scale purchase. Therefore, we estimated the cost-effectiveness with a vaccine price of €10 instead of €18.30 used in the baseline analysis.

As, to our knowledge, only one study explicitly measured the decrease of quality of life due to pertussis disease, we varied this parameter in sensitivity analysis [45]. Here, we assessed the effect on the outcomes if the quality weights were both decreased and increased by 10%.

Furthermore, we investigated the effect of varying work loss on the cost-effectiveness. Firstly, we estimated the effect of a 10% increase and 10% decrease on the results. Furthermore, we assessed the cost-effectiveness when the indirect costs were fully omitted from the analysis.

Finally, we explored the impact of different discount rates on the outcomes. In particular, health outcomes were discounted at a rate of 4% according to the former Dutch guidelines for pharmacoeconomic research [48]. Furthermore, the effect of no discounting for both costs and health outcomes was investigated.

## Results

**Figure 2:**
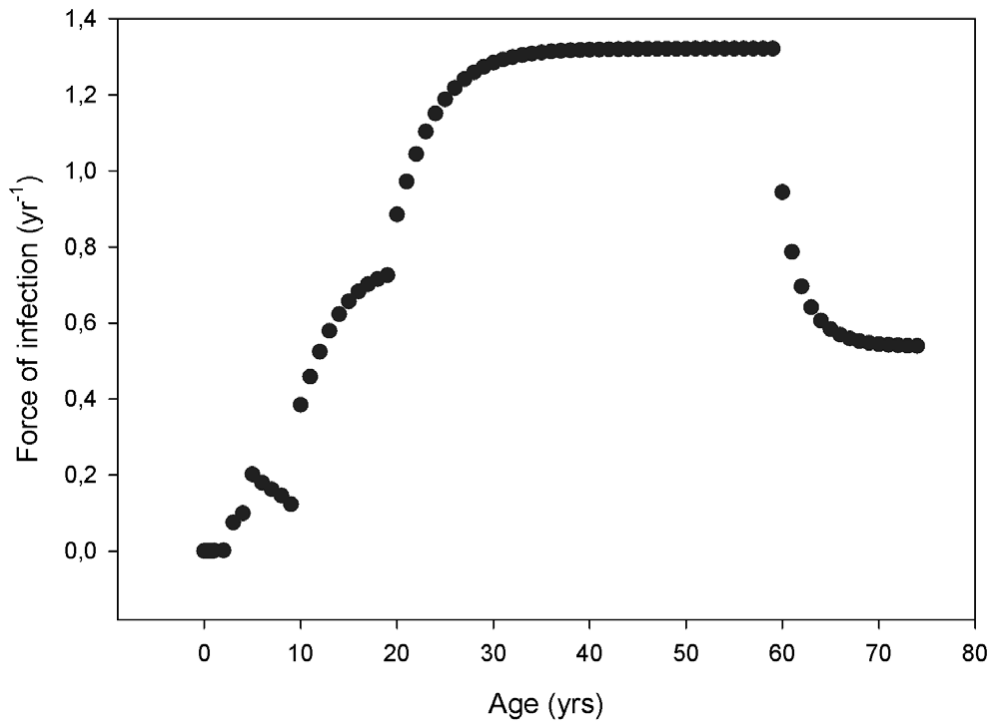
Age-dependent force of infection (FOI) estimated from the Dutch 1996–2000 pertussis incidence data.

Figure 2 shows the estimated age-dependent FOI. The FOI is estimated to be low in the very young age classes and it reaches a peak in adults after which it decreases again in the oldest age classes. This indicates that adults are at the highest risk of acquiring a pertussis infection. Quantitatively, as the period of full immunity after either vaccination or natural infection is similar in both scenarios (i.e. 2 years), the age-dependent FOI is equal for both scenarios investigated.

**Figure 3:**
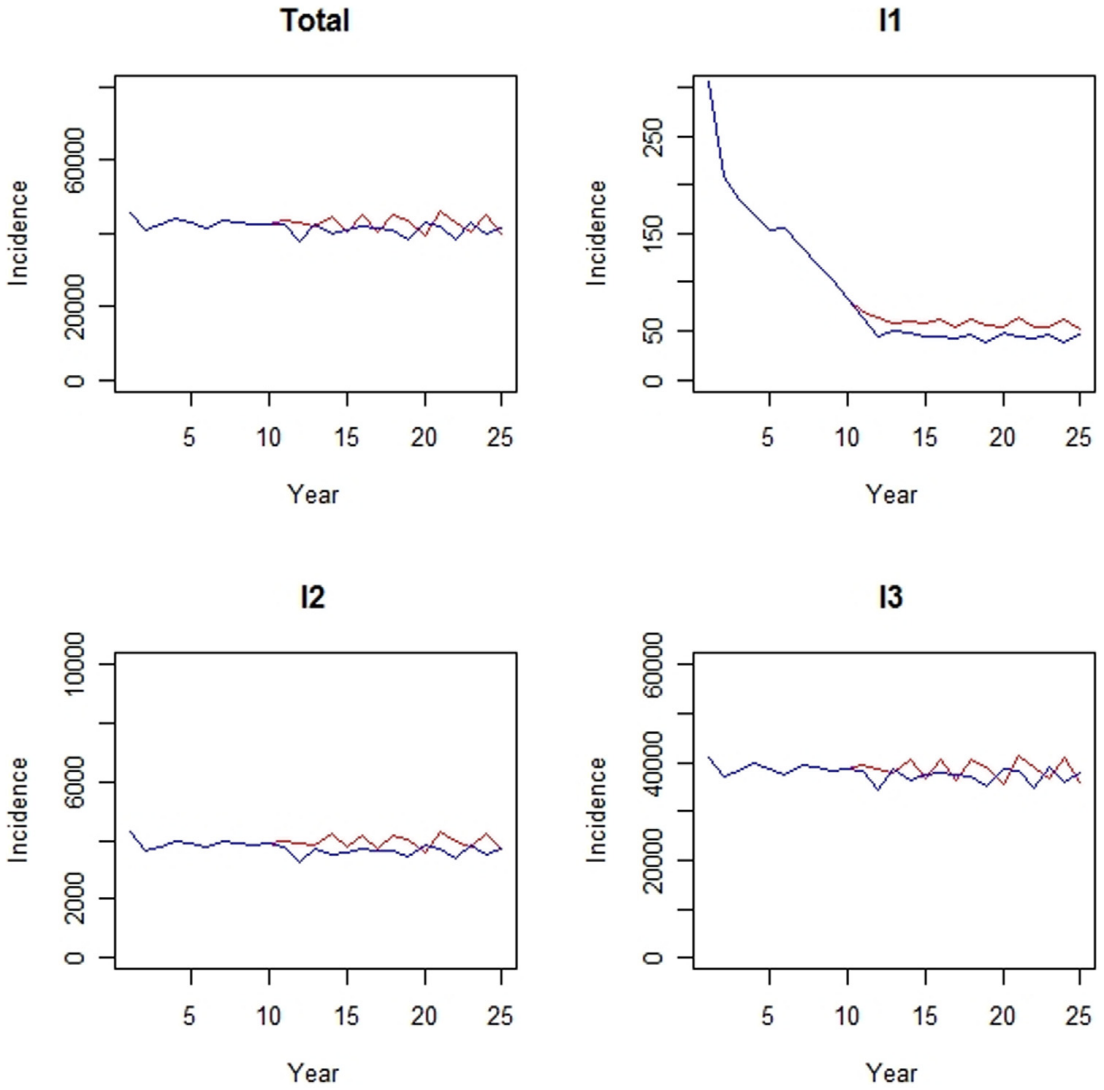
Absolute number of pertussis cases (incidence) for a population 150.000 persons after implementation of booster vaccination at the age of 4 years (t = 0) assuming natural immunity wanes after 15 years. The situation with adolescent vaccination at t = 10 and the current situation without adolescent vaccination are represented by the red and blue lines, respectively. I_1_ = primary infection, I_2_ = recidive infection, I_3_ = asymptomatic infection and Total = I_1_+I_2_+I_3_. Note the different scales of the y-axis for each infection type.

The implementation of an adolescent pertussis booster vaccination program (technically, at t = 10) reduces the absolute incidence of all infection types (I_1_, I_1_ and I_3_) in the total population as shown in Figure 3. In particular, the figure represents the mean outcomes for the scenario where natural immunity is assumed to wane over 15 years. The decreasing trend in incidence is also observed for the other scenario with 8 years of protection (not shown). Note that although the overall incidence in the population was equal in the steady situation for both scenarios, the difference in duration of immunity led to different steady state ratios of I_1_∶I_2_∶I_3_ (i.e. the ratio of symptomatic versus asymptomatic cases differed in both scenarios). The relative decrease in incidence is most apparent for primary pertussis infections. Furthermore, note the relative large decrease in primary infections due to the booster vaccination at the age of 4 years (technically, implemented at t = 0). The age-specific outcomes per infection type are shown in Table 5. Highest percentage reductions in symptomatic infections (i.e. I_1_ and I_2_) are seen in the adolescent age groups (i.e. 10–19 years) and in younger age groups (i.e. 4–9 years) for I_3_. For infants aged below 1 year, 10–20% of primary infections is estimated to be averted. Although the total number of recidive infections declines due to adolescent booster vaccination, the incidence in the older age classes increases slightly. Ergo, adolescent vaccination causes a relative slight age shift for recidive infections.

**Table 5:** Mean differences* in absolute incidence numbers between the current situation and the situation with adolescent pertussis booster vaccination over a 25 year period for a population of 150,000 people (percentage reductions between brackets).

Age	R_n_ = 8 years			R_n_ = 15 years		
	I_1_	I_2_	I_3_	I_1_	I_2_	I_3_
0 yr	3 (12%)	≈0	≈0	4 (18%)	≈0	≈0
1–3 yrs	10 (3%)	19 (5%)	55 (6%)	17 (10%)	22 (6%)	74 (8%)
4–9 yrs	33 (12%)	476 (14%)	1283 (15%)	39 (14%)	523 (17%)	1555 (19%)
10–19 yrs	140 (41%)	7455 (29%)	6540 (11%)	143 (41%)	6040 (32%)	9436 (14%)
20–49 yrs	≈0	−1185 (−3%)	6752 (2%)	≈0	−1016 (−5%)	7646 (3%)
50–74 yrs	0**	−385 (−1%)	4158 (2%)	0**	−372 (−2%)	5354 (3%)

R_n_ = loss of immunity after natural infection.

I_1_ = primary infection, I_2_ = recidive infection and I_3_ = asymptomatic infection.

≈0 refers to the situation where: −1.00<incidence<1.00.

Negative numbers indicate an increase in incidence numbers as a result of adolescent vaccination.

*Due to the extensive running time of the model―related to the high complexity―only 20 runs per scenario were performed.

**No I_1_ exists anymore beyond 50 years of age.

The mean age-specific outcomes as a result of the implementation of an adolescent pertussis vaccination program are shown in Table 6, in terms of costs and health gains. The indirect costs due to productivity losses comprise by far the largest part of the costs associated with pertussis diseases. Specifically, the indirect costs show an increase in the older age groups (i.e. age ≥20 years) as a result of the adolescent pertussis vaccination. Furthermore, the vaccination costs over a 15-year period are estimated at €540.000. Consequently, the incremental cost-effectiveness of adolescent pertussis vaccination is estimated at €4418/QALY (range: 3205–6364 € per QALY) and €6371/QALY (range: 4139–9549 € per QALY) for the scenario with 8 and 15 years protection after natural infection, respectively.

**Table 6:** Mean age-specific outcomes* in terms of costs and QALYs. Absolute differences between current situation and the situation with adolescent pertussis booster vaccination over the 25 year period for a population of 150,000 people.

Age	R_n_ = 8 years			R_n_ = 15 years		
	Direct medical costs (€)	Indirect costs (€)	QALYs	Direct medical costs (€)	Indirect costs (€)	QALYs
*0 yr*	10,786	1612	0.23	12,378	1651	0.41
*1–3 yrs*	601	4613	1.04	1586	8380	1.84
*4–9 yrs*	669	77,474	8.86	740	86,645	9.83
*10–19 yrs*	737	275,300	137.76	780	138,976	112.25
*20–49 yrs*	−127	−281,796	−25.23	−48	−271,117	−21.21
*50–74 yrs*	−42	−56,864	−8.00	−77	−48,103	−7.74
*Total*	12,624	20,339	114.65	15,360	−83,568	95.38

Negative numbers indicate an increase in costs or a decrease in QALYs as a result of adolescent vaccination.

*Due to the extensive running time of the model―related to the high complexity―only 20 runs per scenario were performed.

R_n_ = loss of immunity after natural infection.

Finally, table 7 presents the results of the univariate sensitivity analysis. Except a 10% increase in quality weights, changes adopted in the cost- en health-related parameters had limited effect on the cost-effectiveness.

**Table 7:** Outcomes of the univariate sensitivity analysis.

	R_n_ = 8 years	R_n_ = 15 years
	ICER (costs/QALY)	ICER (costs/QALY)
Baseline Analysis	4418	6371
Vaccine price €10	1649	3043
Quality weights −10%	2338	3387
Quality weights +10%	31,797	40,955
Productivity losses −10%	4436	6284
Productivity losses +10%	4400	6459
No productivity losses	4595	5495
Discount rate health 4%	5224	7415
No discounting	5162	7887

R_n_ = loss of immunity after natural infection.

ICER = incremental cost-effectiveness ratio.

QALY = quality adjusted life year.

## Discussion

In this paper we present the first dynamic individual-based approach to model pertussis transmission in the population. Furthermore, we applied this model to estimate the cost-effectiveness of a universal adolescent booster vaccination program for the Dutch situation. Until now, almost all cost-effectiveness analyses of pertussis vaccination programs previously performed use standard decision analytic models to assess the effects of vaccination [40], [46], [49]–[62]. However, as pertussis is a transmissible infectious disease, a dynamic model is required to fully take into account the spread of the disease over time in the population [20], [63]. Although van Boven et al [23], [64] and Hethcote [65]–[69] used a dynamic approach to model pertussis transmission, these models were not used for economic analyses. So far, only Edmunds et al [70] designed a dynamic model to estimate the cost-effectiveness of pertussis booster vaccination in England and Wales. Our model differs from the dynamic models designed by van Boven, Hethcote and Edmunds in that those models are deterministic and population-based whereas ours is stochastic and individual-based [23], [64]–[70].

In general, reliable and currently valid pertussis related FOI estimates are difficult to obtain. Previously used estimates are mostly based on incidence data from England and Wales before the introduction of widespread vaccination [20], [65], [71]. These estimates may however not describe the current situation in the Netherlands. Moreover the methods used to estimate these age-specific FOIs has a number of drawbacks as described by van Boven et al [23]. As van Boven et al attempted to overcome these drawbacks in their approach, we used the method they [23] developed to consistently take the waning of immunity and different types of infection into account to estimate age-specific FOIs for the Dutch situation. We note that, unlike the stochastic individual-based dynamic framework to model the pertussis transmission in the population, the approach used to estimate the age-specific FOIs was deterministic. However, as the structure of both models and the parameter values used, after converting from rates to probabilities, were similar, we felt this approach could be justified.

The FOI estimated in this study differs from those previously estimated for the Netherlands and England and Wales in terms of both magnitude and shape [20], [23], [65], [71]. The age-specific FOIs estimated in this study are consistently higher than those estimated by Grenfell and Anderson [71] and van Boven et al [23]. This is due to the fact that we, contrary to others, corrected the incidence numbers for occurrence of asymptomatic infections and underreporting. Consequently, the number of infectious people in the population dramatically increased, resulting in an increased rate at which susceptibles would acquire infection. Furthermore, the difference in shape can be explained by different levels of underreporting for different age-classes [33]. In particular, the underreporting rate is much higher in the older age-classes compared to infants.

According our DES-model, the incremental cost-effectiveness of adolescent pertussis booster vaccination is estimated below €10,000 per QALY for both scenarios investigated and in a wide range of sensitivity analyses. The cost-effectiveness would be slightly more favorable if the duration of immunity after natural infection is 8 years (ICER: €4418 per QALY) compared to 15 years (ICER: €6371 per QALY). This is mainly due to the greater amount of QALYs that can be gained by vaccination when the duration of immunity after natural infection is “only” 8 years. Obviously, the shorter the duration of immunity after natural infection (i.e. the faster one becomes susceptible again), the higher the risk of acquiring a symptomatic infection will be. In particular, more recidive infections and associated quality of life loss can be averted in the individuals aged 10–19 years old.

Although adolescent vaccination leads to a decrease in total incidence of pertussis in the population, it caused an increase in absolute numbers of recidive infections in the older age groups (>20 years). Consequently, the total indirect costs increased as a result of adolescent vaccination because the productivity losses are highest in these age classes. Furthermore, the direct medical costs also increased in adults due to the increase in recidive infections in these individuals. This particularly stresses the importance of using a dynamic approach instead of a static one when estimating the cost-effectiveness of pertussis booster vaccination strategies as static models are not able to predict these age shifts. Obviously, the age shift modeled here slightly worsens the cost-effectiveness of adolescent vaccination. Although it is often thought that the inclusion of herd-immunity effects can only have beneficial effects, they can be detrimental as well, as previously also noticed by Brisson et al [72].

Here, we will discuss a possible explanation for the increase in recidive infections in adults. First, the total incidence in the vaccinated age groups will decrease as a result of vaccination. Subsequently, this will lead to a decrease of the infection pressure in the population (i.e. the probability of contacting an infectious individual will decrease). Ergo, the probability of becoming susceptible again will increase for individuals. Consequently, the ratio of symptomatic versus asymptomatic cases will change in favor of the symptomatic cases. Accordingly, although the total incidence decreased, in this analysis, under these baseline assumptions, this resulted in an absolute increase of symptomatic cases in the adult groups. However, the exact effects of adolescent vaccination on the relative and absolute incidences will obviously depend on the underlying assumptions. For example, assuming no infectiousness for asymptomatically infected persons could possibly result in an unintended increase of incidence in young infants if the absolute number of symptomatic cases increases in adults. However, on the contrary, the cost-effectiveness could possibly become more favorable if the infectiousness of asymptomatic cases is increased relatively to that of symptomatic cases. This is certainly an area for further research.

Univariate sensitivity analyses revealed that varying discount rates and parameters associated with cost measures had limited effect on the cost-effectiveness. Regarding quality of life measures only an increase of 10% in the quality weights for pertussis illness showed a considerable effect (i.e. increase) on the ICER. However, such a large increase in quality weights would be rather unrealistic as the increased weights, especially in adolescents and adults, approach one (i.e. perfect health) which would mean that pertussis illness would hardly influence the quality of life of an individual.

All previously performed cost-effectiveness analyses using a static approach showed potential favourable cost-effectiveness for adolescent pertussis booster vaccination [49], [59], [60], [61], [73]. Iskedjian et al [59] even estimated that vaccination of 14-year old adolescents in Ontario (Canada) could be a cost-saving strategy from a societal viewpoint. However, these studies were not able to fully capture the herd-immunity effects as that would require a dynamic approach. The only cost-effectiveness analysis based on a dynamic model, performed by Edmunds et al [70], reported more reserved conclusions. In particular, they estimated that the introduction of a booster at 4 years is possibly more cost-effective than the implementation of an adolescent vaccination program in the UK [70]. This immediately stresses another difficulty in comparing the outcomes of different cost-effectiveness analyses. The effects of adolescent booster vaccination on the epidemiology and cost-effectiveness will differ among different countries as there is a lot of variability in national vaccination policies [1], [74]. For example, in the Netherlands booster vaccination for 4-year olds was indeed actually recently introduced and was present in all our strategies, rather than serving as an alternative. Furthermore, it depends on the exact organization of the current national immunization schedule what will be the best timing of vaccinating adolescents.

There are several limitations of our study. The main disadvantage of the current version of the model is the running time. Although ideally one wants, to fully capture all variability in the outcomes, to run a stochastic model many (e.g. 10,000) times, this was not fully possible here within a reasonable time. Consequently, we were able to run the model only a few times. However, this limited number of replications also already shows insight in the uncertainty surrounding the cost-effectiveness. In this situation (i.e. 20 runs per scenario) the incremental cost-effectiveness never exceeded the €10,000 per QALY for both scenarios which makes it plausible that the expected cost-effectiveness is very likely below this value.

Also, deaths from pertussis were conservatively not taken into account as on average less than one death per year was reported over the last decade in the Netherlands [44]. However, from other countries we do know that there is potential underreporting of pertussis related deaths [75], [76]. Although different national surveillance systems are often not comparable, underreporting of deaths probably also exists in the Netherlands. This is because of the difficulty in defining pertussis as the cause of death. People often decease as a result of the complications (e.g. pneumonia) which will be reported as the cause of death, with the underlying pertussis disease being missed. The inclusion of pertussis related deaths would probably lead to a slightly more favourable cost-effectiveness as adolescent vaccination is estimated to slightly decrease the incidence in the most vulnerable group (i.e. young infants). However, if, on the other hand, unreported deaths more often occur in adults, the inclusion of this underreporting in mortality could lead to less favourable cost-effectiveness

Furthermore, we did not take side effects of the booster vaccine into account. However, the inclusion of side effects will probably not result in a significant increase in the cost-effectiveness as acellular pertussis vaccines for adolescents are generally characterized as safe [29], [31]. In clinical trials no significant differences in systematic symptoms between placebo and vaccine groups were noted [29], [31]. Although local reactions can occur, these would not incur any considerable costs or quality of life loss.

Finally, we used incidence data from 1996–2000 to obtain age-specific steady state incidences, which are required in order to discard the time dependence in the incidence and prevalence. Although we are fully aware of the fact that this period did not reflect an endemic equilibrium in reality, we could not neglect the clear increase in incidence numbers from 1996 onwards. Furthermore, data from 2001 onwards was not included as since the start of that year pertussis booster vaccination for 4-year olds has been introduced in the Netherlands. Ergo, we believe that our approach is most optimal in the current circumstances. Unfortunately we are not able to reliably predict the implications of this assumption on the cost-effectiveness. To do so, the exact cause of the rise in incidence numbers from 1996 has to be known in order to precisely model this increase.

In this analysis we estimated the cost-effectiveness of an acellular pertussis booster vaccine for adolescents. However, in practice the formulation given can also contain diphtheria and tetanus toxoids (dTpa vaccine) [77]. The cost-effectiveness of this combination vaccine could differ from the cost-effectiveness of a single acellular pertussis booster vaccine. Whether or not this combination vaccine will be more cost-effective depends on whether the extra costs outweigh the extra benefits by preventing diphtheria and tetanus as well. To fully and appropriately evaluate the health economic consequences of this combination vaccine all three diseases should be taken into account in the model.

Ideally, a health-economic model is generalisable from one country to the other. Our model can be applied to other countries, however several complicating factors have to be taken into account. In particular, as our model is for an infectious disease, local and national transmission patterns are of importance and the model should be adapted accordingly to such patterns that will differ from the Dutch situation applied in this analysis. Furthermore, national vaccination schedules and exact types of pertussis vaccines used may differ, for example, regarding the number of components included, specific brands and formulations. Obviously, efficacies of different schedules, combinations and brands may differ. For example, Jefferson et al [27] showed that the vaccine effectiveness of vaccines measured in children varied considerably from manufacturer to manufacturer. Taking such differences between countries adequately into account would allow our modelling to be extended to other countries. Initiatives for such further work are indeed currently undertaken.

### Conclusion

In conclusion, we designed a dynamic individual-based DES-model to model pertussis transmission in the population in order to estimate the cost-effectiveness of universal booster vaccination strategies. As also holds true for other infectious diseases, to appropriately asses the cost-effectiveness of pertussis vaccination inclusion of herd-immunity effects is crucial as vaccination considerably reduces the transmission in the population. We have shown that adolescent booster vaccination is likely to be a cost-effective intervention for the Dutch situation, as in the Netherlands interventions are certainly considered cost-effective if cost-effectiveness is estimated below a threshold of € 20,000 per QALY [78]. However, to make a through-out decision about the implementation of such a pertussis booster vaccination strategy, one should first compare the cost-effectiveness of the adolescent vaccination with that of other vaccination strategies (e.g. adult vaccination or cocooning). This further stresses the reason for developing an individual based model. As a next step a social network can be easily included to appropriately model household contacts in order to accurately estimate the cost-effectiveness of a cocooning strategy. Currently, mixing is only based on age. The main disadvantage of the model is the considerable running time, which made it impossible to run hundreds of simulations. Currently, we are programming the model in another computer language in order to try to speed up the modeling process.

## Supporting Information

Appendix S1 (0.15 MB DOC)
