# Supplementary material for: Cost-Effectiveness of Adolescent Pertussis Vaccination for The Netherlands: Using an Individual-Based Dynamic Model
Source: PLoS One. 2010 Oct 15;5(10):e13392. doi: 10.1371/journal.pone.0013392 (PMC2955521; doi:10.1371/journal.pone.0013392)
Supplement: Appendix S1 — (0.15 MB DOC) [file pone.0013392.s001.doc]

Appendix A: Force of Infection

To obtain estimates of the age-specific force of infection (FOI), we assumed that the disease had reached endemic equilibrium. Furthermore, we assumed that everyone exactly lives to 75 years of age. Subsequently, we used the following set of ordinary differential equations to estimate the age specific FOI:

$$\frac{\text{dS}_{1}(a)}{\text{d}a}=-\lambda(a)S_{1}(a)-v(a)S_{1}(a)$$

$$\frac{\text{dI}_{1}(a)}{\text{d}a}=\lambda(a)S_{1}(a)-\rho_{1}I_{1}(a)$$

$$\frac{\text{dR}_{\text{n1}}(a)}{\text{d}a}=\rho_{1}I_{1}(a)+\rho_{2}I_{2}(a)+\rho_{3}I_{3}(a)-\sigma_{n1}R_{n1}(a)$$

$$\frac{\text{dR}_{\text{n2}}(a)}{\text{d}a}=\sigma_{n1}R_{n1}(a)-\sigma_{n2}R_{n2}(a)-\lambda(a)R_{n2}(a)$$

$$\frac{\text{dR}_{\text{v1}}(a)}{\text{d}a}=v(a)S_{1}(a)-\sigma_{v1}R_{v1}(a)$$

$$\frac{\text{dR}_{\text{v2}}(a)}{\text{d}a}=\sigma_{v1}R_{v1}(a)-\sigma_{v2}R_{v2}(a)-\lambda(a)R_{v2}(a)$$

$$\frac{\text{dS}_{2}(a)}{\text{d}a}=\sigma_{n2}R_{n2}(a)+\sigma_{v2}R_{v2}(a)-\lambda(a)S_{2}(a)$$

$$\frac{\text{dI}_{2}(a)}{\text{d}a}=\lambda(a)S_{2}(a)-\rho_{2}I_{2}(a)$$

$$\frac{\text{dI}_{3}(a)}{\text{d}a}=\lambda(a)R_{n2}(a)+\lambda(a)R_{v2}(a)-\rho_{3}I_{3}(a)$$

The epidemiologic parameters and their values in these equations are given in Table A1. We used the mean Dutch age-specific incidence, corrected for underreporting, over the period 1996 until 2000 to estimate the age-specific FOI. Note that for the estimation of the FOI only the vaccination within the first year of life was included. Furthermore, these differential equations were only used for estimation of the age-specific FOI as the pertussis transmission was modelled stochastically. For a complete description of the methodology to estimate the age-specific FOI we refer to van Boven et al [23].

Appendix B: Notification Rates

The age- and infection-type-specific notification rates are shown in table B1. Note that the reporting rates vary for both scenarios as the steady state distributions (I_1_:I_2_:I_3_) differ.

**Appendix C: Hospitalization Rates and Accompanying Length of Stay**

The age- and infection-type-specific hospitalization rates are shown in Table C1. To obtain sufficient numbers for reliably estimating the hospitalization rates we divided the population in 6 different age classes (i.e. 0 yr, 1-3 yrs, 4-9 yrs, 10-19 yrs, 20-59 yrs and 60+ yrs). Note that the hospitalization rates vary for both scenarios as the steady state distributions (I_1_:I_2_:I_3_) differ.

Furthermore, the age specific mean lengths of stay for both standard and intensive care are shown in Table C2. Here we divided the “1-3 years” age group in separate year classes as there were no pertussis related intensive care admissions registered for 2- and 3-year old infants.

Finally, the assumed age-specific fractions of notified cases treated ambulatory by a medical specialist are given in Table C3.

Table A1. Epidemiologic parameter values used for estimation of the age-specific force of infection.

| Parameter | Symbol | Value S1 (S2)^#^ |
| --- | --- | --- |
| Density of susceptible individuals | S_1_(a) | Age dependent |
| Density of susceptible individuals after loss of immunity | S_2_(a) | Age dependent |
| Density of primary infected individuals | I_1_(a) | Age dependent |
| Density of recidive infected individuals | I_2_(a) | Age dependent |
| Density of asymptomatic infected individuals | I_3_(a) | Age dependent |
| Density of recovered and fully immune individuals after infection | R_n1_(a) | Age dependent |
| Density of recovered and partially immune individuals after infection | R_n2_(a) | Age dependent |
| Density of recovered and fully immune individuals after vaccination | R_v1_(a) | Age dependent |
| Density of recovered and partially immune individuals after vaccination | R_v2_(a) | Age dependent |
| Force of infection | λ(a) | Age dependent |
| Vaccination function^*^ | ν(a) | Age dependent |
| Recovery rate after primary infection | ρ_1_ | 13 |
| Recovery rate after recidive infection | ρ_2_ | 17.33 |
| Recovery rate asymptomatic infection | ρ_3_ | 52 |
| Loss of full immunity rate after infection | σ_n1_ | 0.50 (0.50) |
| Loss of partial immunity rate after infection | σ_n2_ | 0.08 (0.17) |
| Loss of full immunity rate after vaccination | σ_v1_ | 0.50 (0.50) |
| Loss of partial immunity rate after vaccination | σ_v2_ | 0.17 (0.17) |

a = age.

S = scenario (see ‘Methods’ section for a definition of both scenarios).

^#^ = yearly rates.

^*^We assumed that a certain fraction of the population (coverage × efficacy) is effectively protected precisely after 4 months.

Table B1. Age- and infection-type-specific notification rates.

| Age (yr) | Scenario: R_n_ = 8 yr | | | Scenario: R_n_ = 15 yr | | |
| --- | --- | --- | --- | --- | --- | --- |
|  | I_1_ | I_2_ | I_3_ | I_1_ | I_2_ | I_3_ |
| *0* | 1.0000 | 1.0000 | 0.0000 | 1.0000 | 1.0000 | 0.0000 |
| *1/12* | 1.0000 | 1.0000 | 0.0000 | 1.0000 | 1.0000 | 0.0000 |
| *2/12* | 1.0000 | 1.0000 | 0.0000 | 1.0000 | 1.0000 | 0.0000 |
| *3/12* | 1.0000 | 1.0000 | 0.0000 | 1.0000 | 1.0000 | 0.0000 |
| *4/12* | 1.0000 | 1.0000 | 0.0000 | 1.0000 | 1.0000 | 0.0000 |
| *5/12* | 1.0000 | 1.0000 | 0.0000 | 1.0000 | 1.0000 | 0.0000 |
| *6/12* | 1.0000 | 1.0000 | 0.0000 | 1.0000 | 1.0000 | 0.0000 |
| *7/12* | 1.0000 | 1.0000 | 0.0000 | 1.0000 | 1.0000 | 0.0000 |
| *8/12* | 1.0000 | 1.0000 | 0.0000 | 1.0000 | 1.0000 | 0.0000 |
| *9/12* | 1.0000 | 1.0000 | 0.0000 | 1.0000 | 1.0000 | 0.0000 |
| *10/12* | 1.0000 | 1.0000 | 0.0000 | 1.0000 | 1.0000 | 0.0000 |
| *11/12* | 1.0000 | 1.0000 | 0.0000 | 1.0000 | 1.0000 | 0.0000 |
| *1* | 1.0000 | 1.0000 | 0.0000 | 1.0000 | 1.0000 | 0.0000 |
| *2* | 1.0000 | 1.0000 | 0.0000 | 1.0000 | 1.0000 | 0.0000 |
| *3* | 0.1511 | 0.0000 | 0.0000 | 0.1511 | 0.0000 | 0.0000 |
| *4* | 0.1696 | 0.0000 | 0.0000 | 0.1696 | 0.0000 | 0.0000 |
| *5* | 0.0838 | 0.0000 | 0.0000 | 0.0838 | 0.0000 | 0.0000 |
| *6* | 0.0971 | 0.0000 | 0.0000 | 0.0971 | 0.0000 | 0.0000 |
| *7* | 0.1143 | 0.0000 | 0.0000 | 0.1143 | 0.0000 | 0.0000 |
| *8* | 0.1345 | 0.0000 | 0.0000 | 0.1345 | 0.0000 | 0.0000 |
| *9* | 0.1569 | 0.0000 | 0.0000 | 0.1569 | 0.0000 | 0.0000 |
| *10* | 0.0170 | 0.0000 | 0.0000 | 0.0170 | 0.0000 | 0.0000 |
| *11* | 0.0221 | 0.0000 | 0.0000 | 0.0221 | 0.0000 | 0.0000 |
| *12* | 0.0321 | 0.0000 | 0.0000 | 0.0321 | 0.0000 | 0.0000 |
| *13* | 0.0511 | 0.0000 | 0.0000 | 0.0511 | 0.0000 | 0.0000 |
| *14* | 0.0875 | 0.0000 | 0.0000 | 0.0876 | 0.0000 | 0.0000 |
| *15* | 0.1584 | 0.0000 | 0.0000 | 0.1586 | 0.0000 | 0.0000 |
| *16* | 0.2993 | 0.0000 | 0.0000 | 0.2997 | 0.0000 | 0.0000 |
| *17* | 0.5838 | 0.0000 | 0.0000 | 0.5844 | 0.0000 | 0.0000 |
| *18* | 1.0000 | 0.0008 | 0.0000 | 1.0000 | 0.0015 | 0.0000 |
| *19* | 1.0000 | 0.0032 | 0.0000 | 1.0000 | 0.0061 | 0.0000 |
| *20* | 0.9879 | 0.0000 | 0.0000 | 0.9890 | 0.0000 | 0.0000 |
| *21* | 1.0000 | 0.0007 | 0.0000 | 1.0000 | 0.0014 | 0.0000 |
| *22* | 1.0000 | 0.0012 | 0.0000 | 1.0000 | 0.0023 | 0.0000 |
| *23* | 1.0000 | 0.0014 | 0.0000 | 1.0000 | 0.0028 | 0.0000 |
| *24* | 1.0000 | 0.0016 | 0.0000 | 1.0000 | 0.0031 | 0.0000 |
| *25* | 1.0000 | 0.0017 | 0.0000 | 1.0000 | 0.0033 | 0.0000 |
| *26* | 1.0000 | 0.0017 | 0.0000 | 1.0000 | 0.0035 | 0.0000 |
| *27* | 1.0000 | 0.0018 | 0.0000 | 1.0000 | 0.0036 | 0.0000 |
| *28* | 1.0000 | 0.0018 | 0.0000 | 1.0000 | 0.0037 | 0.0000 |
| *29* | 1.0000 | 0.0018 | 0.0000 | 1.0000 | 0.0037 | 0.0000 |
| *30* | 1.0000 | 0.0019 | 0.0000 | 1.0000 | 0.0038 | 0.0000 |
| *31* | 1.0000 | 0.0019 | 0.0000 | 1.0000 | 0.0038 | 0.0000 |
| *32* | 1.0000 | 0.0019 | 0.0000 | 1.0000 | 0.0039 | 0.0000 |
| *33* | 1.0000 | 0.0019 | 0.0000 | 1.0000 | 0.0039 | 0.0000 |
| *34* | 1.0000 | 0.0019 | 0.0000 | 1.0000 | 0.0039 | 0.0000 |
| *35* | 1.0000 | 0.0019 | 0.0000 | 1.0000 | 0.0039 | 0.0000 |
| *36* | 1.0000 | 0.0019 | 0.0000 | 1.0000 | 0.0039 | 0.0000 |
| *37* | 1.0000 | 0.0019 | 0.0000 | 1.0000 | 0.0039 | 0.0000 |
| *38* | 1.0000 | 0.0019 | 0.0000 | 1.0000 | 0.0039 | 0.0000 |
| *39* | 1.0000 | 0.0019 | 0.0000 | 1.0000 | 0.0039 | 0.0000 |
| *40* | 1.0000 | 0.0019 | 0.0000 | 1.0000 | 0.0039 | 0.0000 |
| *41* | 1.0000 | 0.0019 | 0.0000 | 1.0000 | 0.0039 | 0.0000 |
| *42* | 1.0000 | 0.0019 | 0.0000 | 1.0000 | 0.0039 | 0.0000 |
| *43* | 1.0000 | 0.0019 | 0.0000 | 1.0000 | 0.0039 | 0.0000 |
| *44* | 1.0000 | 0.0019 | 0.0000 | 1.0000 | 0.0039 | 0.0000 |
| *45* | 1.0000 | 0.0019 | 0.0000 | 1.0000 | 0.0039 | 0.0000 |
| *46* | 1.0000 | 0.0019 | 0.0000 | 1.0000 | 0.0039 | 0.0000 |
| *47* | 1.0000 | 0.0019 | 0.0000 | 1.0000 | 0.0039 | 0.0000 |
| *48* | 1.0000 | 0.0019 | 0.0000 | 1.0000 | 0.0039 | 0.0000 |
| *49* | 1.0000 | 0.0019 | 0.0000 | 1.0000 | 0.0039 | 0.0000 |
| *50* | 1.0000 | 0.0019 | 0.0000 | 1.0000 | 0.0039 | 0.0000 |
| *51* | 1.0000 | 0.0019 | 0.0000 | 1.0000 | 0.0039 | 0.0000 |
| *52* | 1.0000 | 0.0019 | 0.0000 | 1.0000 | 0.0039 | 0.0000 |
| *53* | 1.0000 | 0.0019 | 0.0000 | 1.0000 | 0.0039 | 0.0000 |
| *54* | 1.0000 | 0.0019 | 0.0000 | 1.0000 | 0.0039 | 0.0000 |
| *55* | 1.0000 | 0.0019 | 0.0000 | 1.0000 | 0.0039 | 0.0000 |
| *56* | 1.0000 | 0.0019 | 0.0000 | 1.0000 | 0.0039 | 0.0000 |
| *57* | 1.0000 | 0.0019 | 0.0000 | 1.0000 | 0.0039 | 0.0000 |
| *58* | 1.0000 | 0.0019 | 0.0000 | 1.0000 | 0.0039 | 0.0000 |
| *59* | 1.0000 | 0.0019 | 0.0000 | 1.0000 | 0.0039 | 0.0000 |
| *60* | 1.0000 | 0.0019 | 0.0000 | 1.0000 | 0.0039 | 0.0000 |
| *61* | 1.0000 | 0.0017 | 0.0000 | 1.0000 | 0.0034 | 0.0000 |
| *62* | 1.0000 | 0.0015 | 0.0000 | 1.0000 | 0.0029 | 0.0000 |
| *63* | 1.0000 | 0.0013 | 0.0000 | 1.0000 | 0.0026 | 0.0000 |
| *64* | 1.0000 | 0.0012 | 0.0000 | 1.0000 | 0.0024 | 0.0000 |
| *65* | 1.0000 | 0.0011 | 0.0000 | 1.0000 | 0.0022 | 0.0000 |
| *66* | 1.0000 | 0.0011 | 0.0000 | 1.0000 | 0.0021 | 0.0000 |
| *67* | 1.0000 | 0.0011 | 0.0000 | 1.0000 | 0.0020 | 0.0000 |
| *68* | 1.0000 | 0.0010 | 0.0000 | 1.0000 | 0.0020 | 0.0000 |
| *69* | 1.0000 | 0.0010 | 0.0000 | 1.0000 | 0.0019 | 0.0000 |
| *70* | 1.0000 | 0.0010 | 0.0000 | 1.0000 | 0.0019 | 0.0000 |
| *71* | 1.0000 | 0.0010 | 0.0000 | 1.0000 | 0.0019 | 0.0000 |
| *72* | 1.0000 | 0.0010 | 0.0000 | 1.0000 | 0.0019 | 0.0000 |
| *73* | 1.0000 | 0.0010 | 0.0000 | 1.0000 | 0.0019 | 0.0000 |
| *74* | 1.0000 | 0.0010 | 0.0000 | 1.0000 | 0.0018 | 0.0000 |

I = type of infection (I_1_ = primary infection, I_2_ = recidive infection, I_3_ = asymptomatic infection).

R_n_ = duration of immunity acquired after natural infection.

Table C1. Age- and infection-type-specific hospitalization rates.

| Age class | Scenario: R_n_ =8 | | | Scenario: R_n_ =15 | | |
| --- | --- | --- | --- | --- | --- | --- |
|  | I_1_ | I_2_ | I_3_ | I_1_ | I_2_ | I_3_ |
| *0 yr* | 1.00000 | 1.00000 | 0.00000 | 1.00000 | 1.00000 | 0.00000 |
| *1-3 yrs* | 0.01594 | 0.00000 | 0.00000 | 0.01594 | 0.00000 | 0.00000 |
| *4-9 yrs* | 0.00222 | 0.00000 | 0.00000 | 0.00222 | 0.00000 | 0.00000 |
| *10-19 yrs* | 0.00027 | 0.00000 | 0.00000 | 0.00027 | 0.00000 | 0.00000 |
| *20-59 yrs* | 0.10079 | 0.00000 | 0.00000 | 0.10090 | 0.00000 | 0.00000 |
| *60+ yrs* | 1.00000 | 0.00002 | 0.00000 | 1.00000 | 0.00004 | 0.00000 |

I = type of infection (I_1_ = primary infection, I_2_ = recidive infection, I_3_ = asymptomatic infection).

R_n_ = duration of immunity acquired after natural infection.

Table C2. Age-specific lengths of stay (in days).

| Age class | LOS standard care | LOS intensive care |
| --- | --- | --- |
| *0 yr* | 7.98 | 9.17 |
| *1 yrs* | 3.87 | 10.00 |
| *2 yrs* | 3.80 | - |
| *3 yrs* | 3.36 | - |
| *4-9 yrs* | 4.08 | - |
| *10-19 yrs* | 4.50 | - |
| *20-59 yrs* | 4.70 | - |
| *60+ yrs* | 11.69 | - |

LOS = length of stay.

Table C3. Age-specific probabilities of being treated ambulatory for notified pertussis cases.

| Age class | Fraction treated ambulatory |
| --- | --- |
| *0 yr* | 1 |
| *1-3 yrs* | 0.75 |
| *4-9 yrs* | 0.5 |
| *10-19 yrs* | 0.25 |
| *20-59 yrs* | 0 |
| *60+ yrs* | 0.5 |
